# Use of Electronic Health Records to Develop and Implement a Silent Best Practice Alert Notification System for Patient Recruitment in Clinical Research: Quality Improvement Initiative

**DOI:** 10.2196/10020

**Published:** 2019-04-26

**Authors:** Connor Devoe, Harriett Gabbidon, Nina Schussler, Lauren Cortese, Emily Caplan, Colin Gorman, Kamal Jethwani, Joseph Kvedar, Stephen Agboola

**Affiliations:** 1 Partners HealthCare Pivot Labs Partners HealthCare Boston, MA United States; 2 Harvard Medical School Harvard University Boston, MA United States; 3 Massachusetts General Hospital Boston, MA United States

**Keywords:** recruitment, silent BPA notifications, research, enrollment, innovation, electronic medical record, COPD

## Abstract

**Background:**

Participant recruitment, especially for frail, elderly, hospitalized patients, remains one of the greatest challenges for many research groups. Traditional recruitment methods such as chart reviews are often inefficient, low-yielding, time consuming, and expensive. Best Practice Alert (BPA) systems have previously been used to improve clinical care and inform provider decision making, but the system has not been widely used in the setting of clinical research.

**Objective:**

The primary objective of this quality-improvement initiative was to develop, implement, and refine a silent Best Practice Alert (sBPA) system that could maximize recruitment efficiency.

**Methods:**

The captured duration of the screening sessions for both methods combined with the allotted research coordinator hours in the Emerald-COPD (chronic obstructive pulmonary disease) study budget enabled research coordinators to estimate the cost-efficiency.

**Results:**

Prior to implementation, the sBPA system underwent three primary stages of development. Ultimately, the final iteration produced a system that provided similar results as the manual Epic Reporting Workbench method of screening. A total of 559 potential participants who met the basic prescreen criteria were identified through the two screening methods. Of those, 418 potential participants were identified by both methods simultaneously, 99 were identified only by the Epic Reporting Workbench Method, and 42 were identified only by the sBPA method. Of those identified by the Epic Reporting Workbench, only 12 (of 99, 12.12%) were considered eligible. Of those identified by the sBPA method, 30 (of 42, 71.43%) were considered eligible. Using a side-by-side comparison of the sBPA and the traditional Epic Reporting Workbench method of screening, the sBPA screening method was shown to be approximately four times faster than our previous screening method and estimated a projected 442.5 hours saved over the duration of the study. Additionally, since implementation, the sBPA system identified the equivalent of three additional potential participants per week.

**Conclusions:**

Automation of the recruitment process allowed us to identify potential participants in real time and find more potential participants who meet basic eligibility criteria. sBPA screening is a considerably faster method that allows for more efficient use of resources. This innovative and instrumental functionality can be modified to the needs of other research studies aiming to use the electronic medical records system for participant recruitment.

## Introduction

Although clinical research is critical to our understanding of disease etiology and the development of novel therapeutics, a commonly encountered problem in clinical trials is the challenge of meeting enrollment targets in the stipulated time. For example, a study of neuroimaging in cognitively impaired geriatric patients found that 58% of potential participants failed to enroll due to a lack of interest [1]. Another study that aimed at screening women aged ≥50 years with ovarian cancer demonstrated similar results [2]; only 54.6% of the eligible candidates who were contacted for the study were willing to participate, and those who were not willing to participate cited reasons such as wanting more information from their doctor, inconvenience, or not believing themselves to be at risk for developing ovarian cancer [2]. Among elderly and inpatient populations, this difficulty in recruiting participants is even more apparent. A study that evaluated cognitive dysfunction in older adults after admission for heart failure reported that potential participants expressed interest in participation at the initial encounter, but later rescinded their interest, often without giving a specific explanation [3]. Participants who provided reasons stated they were too tired, too sick, or no longer interested, among others [3]. Given these recruitment challenges, it is imperative to find novel strategies to facilitate participant recruitment in clinical trials.

Electronic health record (EHR) systems have the potential to facilitate rapid patient recruitment in clinical research [4]. This is primarily due to the widespread use of EHRs in clinical practice since the enactment of the Affordable Care Act. Leveraging various functionalities within the EHR system may facilitate earlier patient identification and increased study enrollment. One such EHR functionality is the Best Practice Alert (BPA) notification systems. BPAs are “...automated alerts within the electronic medical record that help facilitate widespread communication of information to primary care providers...” [4]. They are used clinically to save time, identify patients for follow-up, and increase clinician efficiency [5]. In a pilot study at Yale New Haven Hospital, BPAs were used to identify patients that may be good candidates for a smoking-cessation medication [5]. At the San Francisco Medical Center, University of California, one group implemented BPAs in the Apex EHR for the use of telemetry or continuous cardiac monitoring [6]. This BPA notified clinicians about discontinuing the use of telemetry for patients who exceeded the nationally recommended duration for telemetry [7].

One common reason for not adopting BPA is alert fatigue. In one study at the Stanford Medical Center that attempted to integrate BPA into their EHR to provide clinical decision support in the computerized physician order entry for transfusions, it was found that clinicians continued to order transfusion blood products outside of the recommended guidelines, despite BPAs, exposing several questionable practices surrounding transfusions such as perioperative and periprocedural transfusions or orders anticipating imminent discharge [8]. More recently, the Massachusetts General Hospital, Boston, MA, expanded the use of pop-up BPA notifications to alert providers of patients on the opioid registry in the Epic EHR. Through these alerts, patients receiving outpatient prescriptions for opioids can be monitored (Multimedia Appendix 1).

In this quality-improvement project, the study team was interested in taking this framework of using BPA in clinical settings and tailoring it specifically to increase patient recruitment in a clinical trial. To mitigate concerns about alert fatigue, the study staff implemented and tested a “silent” BPA (sBPA) system. The study team defines sBPA as one in which these notifications do not appear as pop-up messages in the EHR view, but rather in a separate inbox or “in-basket” that can be checked periodically. This system eliminates the need to manually search through inpatient admission data by filtering patient data through an algorithm that identifies candidates who meet preselected screening criteria and subsequently sends the list of identified candidate participants to an in-basket messaging service within the EHR. In this project, our goal was to develop an sBPA system that could be used to efficiently identify potential participants admitted to the hospital in order to expedite recruitment in a difficult-to-recruit, elderly inpatient population.

## Methods

### Study Population and Settings

The BPA system used in this quality-improvement project was developed to increase recruitment rates for a prospective observational cohort study (Emerald-COPD study), approved by the Partners HealthCare Institutional Review Board. The study aimed to collect objective measures (eg, physical activity and inhaled medication use) and self-reported subjective measures in patients with chronic obstructive pulmonary disease (COPD). The overall goal of the Emerald-COPD study was to determine whether the collected data could be used to predict the patient’s health status, such as an acute exacerbation of their COPD or readmission due to COPD. The targeted enrollment sample size was 300, and participants were recruited from the inpatient floors of three Partners Healthcare Hospitals: the Massachusetts General Hospital, Brigham and Women’s Hospital, and Brigham and Women’s Faulkner Hospital. The study team consisted of five research coordinators that performed all study procedures. Inclusion criteria included sufficient understanding of the English language, willingness to participate in the research, hospitalization within 24 hours of primary or secondary diagnosis of COPD, and discharge from the hospital to home. In this study, it was important to identify potentially eligible participants prior to hospital discharge as dictated by the study protocol.

### Traditional Recruitment Method, Workflow, and Challenges

The first step in the recruitment process was identification of potentially eligible participants upon hospital admission. Traditionally, this was completed by sorting through the Epic Reporting Workbench module. The module is a reporting tool that lives in the Epic Hyperspace (Partners EHR system) and pulls data from the millions of records in the system into a template form, which includes basic demographic information and admission diagnoses. Research coordinators filtered the form by COPD-related admission diagnoses to reduce the number of records flagged for further review. On an average, research coordinators spent 2 hours per day manually sorting through the Epic Reporting Workbenches of the three recruiting hospitals and reviewing admission notes to determine whether potential participants met the study eligibility criteria. Potential participants who met the basic screening criteria were often excluded for various reasons including unstable psychiatric disposition; illicit drug use; or designation to be discharged to rehab, hospice, or long-term care facilities. Following identification and initial screening, research coordinators secured (via email or page) permission from the care provider as per hospital policy prior to approaching the identified potential participants.

### Overview of the Silent Best Practice Alert System

The Epic EHR system at Partners HealthCare contains an application programming interface that enables seamless integration of programs like the BPA system. For example, in a clinical decision support BPA for medications, the algorithm would search the EHR for potential prescription discrepancies to provide the best possible clinical recommendations. To reduce the time spent screening for potentially eligible participants, the study staff repurposed a similar BPA system to search the EHR and send an alert to research coordinators notifying them that a patient meeting the preselected study criteria was admitted to the hospital, via an in-basket messaging service accessed through the EHR home screen.

The traditional Epic Reporting Workbench lacked a simplified storage system. Study staff would perform daily screenings and create a finalized list of names, associated admitting diagnoses, and care provider contact information in Microsoft Excel. This enabled research coordinators to mark potential candidates as interested or not interested and to fill in the reason for not participating, if the latter applied. The sBPA system provided a solution to this storage problem, as the in-basket could be managed similar to an email inbox. Additionally, the demographic information, including name, admitting diagnosis, and care provider contact information, was included in the alert and stored in the in-basket. This not only simplified the process of identifying potential study candidates, but also facilitated outreach to patients and their providers (Multimedia Appendices 2-5).

#### Development: Iteration I

Study staff and collaborators from Partners eCare Research Core (PeRC) built the sBPA functionality in Epic EHR. The real-time alerts were not in the form of pop-up notifications that led to alert fatigue in the original BPA system; instead, the research coordinators received sBPAs in an email-like, in-basket format. The alerts contained a link to relevant patient information such as a patient’s current medications and past medical history, which further helped the research coordinator in screening for study eligibility.

The preselected criteria provided to the PeRC team fell into the three distinct categories corresponding to established data capture fields in the Epic EHR: admission diagnosis, Epic problem list, and medication list. In this study, the International Statistical Classification of Diseases and Related Health Problems 10th revision (ICD-10) codes of admission diagnoses related to COPD (eg, shortness of breath, dyspnea, cough, pneumonia, and respiratory failure), COPD appearing in problem list, and medications associated with COPD were all included in the set of multiple nested conditions, which were to be met before an alert was triggered. This initial iteration was completed 1 month after providing the preselected criteria to the PeRC team.

#### Implementation

During the initial implementation phase, study staff compared the number of potentially eligible participants provided by the newly implemented sBPA system with our traditional screening method in order to refine the sBPA logic and ensure that all potential participants captured in the manual screening method were also captured through the sBPA. From March to October 2017, weekly comparisons between the two screening methods were assessed for yield of potentially eligible participants and time taken to complete daily screenings. This quality testing was performed by the study research coordinators who used the Epic Workbench and were trained to use the sBPA system. The research coordinators first screened for potential participants through the Epic Reporting Workbench and then screened again using the sBPA. Additionally, both methods were timed to assess the effort required for each screening method. By dividing the cost/hour budgeted for research coordinators’ time by the total time spent using sBPA, the study staff provided an estimate of any projected change in costs, both in hours and US dollars, should a similar system be utilized.

#### Refinement: Iterations II, III, and IV

The original sBPA was setup to flag potential participants who either had an admitting diagnosis related to COPD, COPD in their problem list, or a medication associated with COPD treatment as part of their prescribed inpatient or outpatient medication history. In July 2017, the study staff introduced an iteration of the sBPA by revising the trigger conditions provided to the PeRC team. Instead of flagging potential participants who met at least one of the preselected criteria, the sBPA trigger condition aimed at flagging potential participants who met at least two of the preselected criteria. Again, in early August 2017, the study staff revised the trigger conditions provided to the PeRC team and, with this second iteration, potential participants who were prescribed multiple COPD-related medications during their inpatient stay were not flagged as satisfying two of the three criteria set in the July 2017 iteration.

Based on the results of weekly comparisons during the implementation phase, the sBPA trigger logic was adjusted for a third time in late August 2017 to only capture potential participants who met criteria that fell within two of three preselected categories (eg, patient with COPD in the problem list and COPD-related medication) as opposed to two criteria within the same category (eg, patient with two COPD-related medications). This adjustment was made to account for the number of potential participants flagged by the sBPA who would otherwise not be flagged as eligible in the manual screening method. Another final adjustment was made at the end of the refinement period in October 2017 to strengthen the logic in order to ensure that only potential participants with a hospital inpatient status were flagged as opposed to those who only visited the emergency department but were not transitioned to inpatient admission. This fourth and final iteration added a fourth category of the preselected criteria for the sBPA—inpatient status—yielding the four categories of COPD-related admitting diagnosis, COPD-related medication, COPD in problem list, and inpatient status. Throughout all iterations, the sBPA preselected logic was executed on all hospital admissions across the three participating hospitals.

## Results

**Figure 1:**
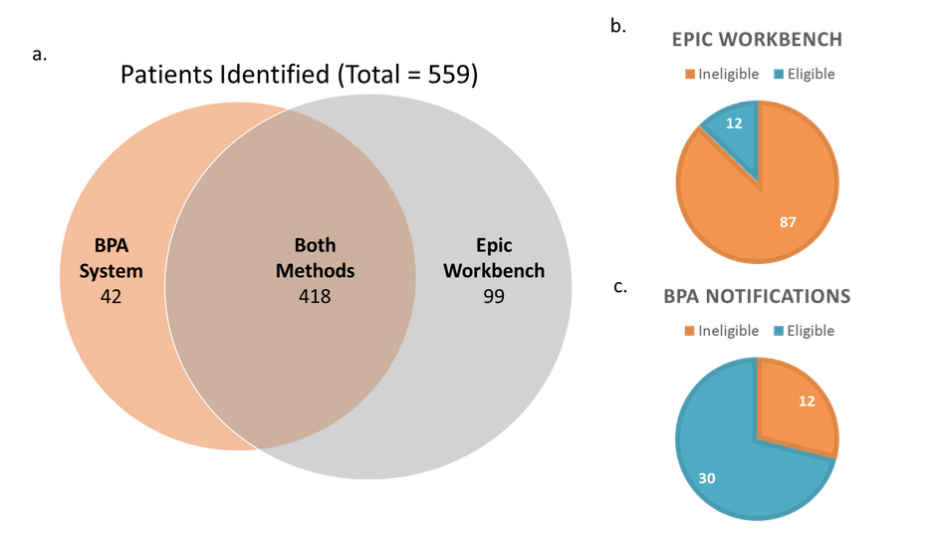
Comparison of identified potential participants: traditional Workbench method versus silent Best Practice Alert. BPA: Best Practice Alert.

Development of the sBPA system was completed by the PeRC team 1 month after the study criteria were received from the study staff. A total of 559 potential participants were identified from March 1 to October 2, 2017, from both screening methods. Of these, 418 (of 559, 74.77%) potential participants were identified by both the Epic Workbench method and sBPA (Figure 1 a). Of the potential participants identified from both screening methods, 287 (of 418, 68.66%) were considered eligible. Of the potential participants considered eligible, 60 participants enrolled in the study. Those who did not enroll either declined or were found to be ineligible for a reason other than that listed in the initial screening criteria (eg, active lung cancer or other serious conditions, psychiatric conditions, or language barrier).

Although the sBPA system was being used simultaneously, the Epic Workbench method found additional 99 potential participants who were not identified via the BPA notifications method (Figure 1 a). Of those found by only the Epic Workbench, 12 potential participants (of 99, 12.12%) were determined to be eligible to participate in the study (Figure 1 b). Over the four iterations, the sBPA notifications method found 42 additional potential participants who were not identified by the Epic Workbench method (Figure 1 a). Of these sBPA-only potential participants, 30 participants (of 42, 71.43%) were determined to be eligible by the emailed physicians (Figure 1 c). In summary, although the sBPA method of screening identified fewer potential participants in total over the course of the project, a greater percentage of the potential participants identified were later confirmed to be eligible for study participation. From the overall increase in identified eligible patients, the study staff determined that the system found the equivalent of three additional eligible potential participants per week.

The sensitivity and specificity of the sBPA system in identifying potential participants varied by iteration. The first iteration of the sBPA system flagged for ICD-10 codes for related admitting diagnoses was related to medications and COPD in the problem list. The initial iteration contained trigger logic that was very sensitive, hence including many potential participants that met at least one of the prespecified conditions, but was not specific enough to identify those who were eligible for study participation. Therefore, during much of the initial phases of development and implementation, there was an overpull of potential participants who did not meet the study requirements. Instead of reducing the time spent screening, the study staff spent more time going through potential participants that had met one of three criteria but were not potentially eligible. To resolve this issue, the study staff introduced the later iterations, which stipulate that individuals must meet two of three requirements. Iteration II yielded many names that lacked specificity. Iteration III appeared to be too restrictive and provided far less names than the earlier iterations. Additionally, Iterations I, II, and III generated many names of individuals who were admitted to the emergency department or were under observation. The study team found that the specificity afforded by the modifications that led to Iteration IV optimized the system to generate names of inpatients who met initial screening requirements.

An additional outcome observed when comparing potential participants missed by the Epic Workbench to the sBPA notifications was that the study staff had missed potentially eligible participants who were not identified by the system because their primary diagnosis was not a COPD-related ICD-10 code (ie, COPD exacerbation, dyspnea, shortness of breath, and chest pain). The Epic Workbench model only filtered information based on the primary admitting diagnosis, and therefore, many individuals were missed if they had a primary admitting diagnosis that did not align with these ICD-10 codes. The specified flagged criteria used by the sBPA system proved to be more effective in identifying eligible participants, because it did not rely solely on these potential participants’ primary diagnoses.

In addition to the increase in the number of identified eligible potential participants, the sBPA system reduced the screening time. The average screening time for the Epic Workbench screening method was 123 minutes per day, and the average time to complete screening with sBPA notifications was 29 minutes per day. Thus, the sBPA notification method was approximately four times faster than the traditional Epic Reporting Workbench method, or yielded a 76.42% decrease in time spent screening. By dividing the cost/hour budgeted for research coordinators’ time by the total time saved with BPA, the cost savings projections, given this increased efficiency, are projected to be US $15,487.50 with over 442.5 hours saved by the end of the study (Table 1). This saving factors in the cost/hour of research coordinators allotted by the study budget (US $35/hour) and the estimated number of hours spent screening over the study duration (approximately 590 hours for the Epic Workbench method and 147.5 hours for the Best Practice Alert notification method).

**Table 1:** Total savings, in hours and US dollars, from the silent Best Practice Alert notifications screening method as compared to the previous method of screening. Expenses are calculated from March 8, 2017, to May 1, 2018 (project completion).

Category	Expenses		
	Cost/hour (US $)	Total hours	Total cost (US $)
Without BPA^a^ notifications	35	590.0	20,650.00
With BPA notifications	35	147.5	5162.50
Saved	N/A^b^	442.5	15,487.50

^a^BPA: Best Practice Alert.

^b^N/A: not applicable.

## Discussion

### Principal Findings

Although BPA notifications are increasingly being used in clinical settings [4-8], this is one of the first studies to implement the application of an sBPA system in the context of clinical research. The primary purpose of this quality-improvement project was to develop, refine, and implement a more efficient and usable version of the BPA system to increase patient recruitment for a clinical trial. Additionally, the study team aimed to determine if automation of the recruitment process through sBPA notifications would not only save time, but also help identify potential participants who were previously missed during our traditional Epic Reporting Workbench screening. Through four iterations, the study team worked to optimize this system through implementation and real-time refinement. Ultimately, sBPA notifications proved to be a considerably faster method of screening. This had a positive impact on the overall success of the study, as a faster recruitment method allowed the study staff to devote more time to other aspects of the research, thus decreasing total hours and, in turn, total cost. Automatic identification of potential participants in real-time through the in-basket reduced the frequency of screening failures and increased the pool of potential participants in a difficult-to-recruit population.

Rapid recruitment and enrollment are vital to the success of any clinical trial, and if the recruitment period must be extended to reach a target sample size, it will delay the trial and result in increased costs [9,10]. This is particularly true for clinical trials involving technology-enabled products, as the spate of innovations in the digital health industry is overwhelming. A device developed a few months prior can become obsolete before the study has had a chance to enroll the targeted enrollment sample size. Nevertheless, digital health solutions, like any other intervention for patient care, need to be rigorously evaluated before broader adoption in clinical settings, and clinical trials remain the gold standard for validating these solutions [9]. Therefore, studies must be designed with methodologies that maintain high internal and external validity and yet allow recruitment to be completed in the shortest time possible. Application of sBPA notifications in clinical studies can facilitate rapid patient enrollment and help study teams meet their recruitment targets.

sBPA notifications have the potential to increase the overall study cost-efficiency by reducing the number of hours the study staff spend on initial patient identification and screening. It is well known that the cost of conducting clinical trials is rapidly increasing, which has negative implications for the development and evaluation of new interventions for patient care [9]. Patient recruitment time accounts for about 30% of the overall study time and is one of the top reasons for the increasing cost of clinical trials [11]. To plan for unavoidable recruitment factors like ineligibility and lack of interest, it is important to efficiently identify as many candidate participants as quickly as possible. In this quality-improvement project, the study team demonstrated that sBPA notifications and other EHR-based methods that facilitate earlier patient identification and screening may increase the cost-efficiency of clinical trials.

Scalability of similar systems across other EHRs for research screening purposes is becoming more possible through initiatives to increase interoperability of these databases across heterogeneous systems. Much of this interoperability is made possible through the utilization of electronic data capture platforms, such as Vanderbilt University’s Research Electronic Data Capture (REDCap) software. Many electronic data capture platforms, including REDCap, have built-in functionalities that facilitate export and import of data from EHRs [12]. In Europe, government initiatives have been implemented to increase shared data across EHR platforms. Electronic Health Records for Clinical Research (EHR4CR) is a €16 million initiative across 35 academic research centers and pharmaceutical companies to create a massive, de-identified EHR data repository in order to assist in prospective eligibility screening and patient recruitment efforts in clinical research [13]. Having secured permissions from patients, the public, and researchers across the continent, the platform also enables researchers to access EHR data from hospitals to determine project feasibility and locate the optimal sites to carry out clinical trials based on their populations [13]. Similar initiatives are seen in the United States, including the Patient-Centered Outcomes Research (PCOR) Institute PCORnet service, a platform that integrates clinical data from 11 clinical data research networks to create sustainable infrastructure for use in comparative effectiveness research [14,15]. These developments are an exciting start; however, more research will be required to assess the functionality of these systems in other modalities such as screening and recruitment.

### Limitations

This project was not designed to test a specific study hypothesis. Therefore, some of the project processes are not predefined or do not adhere to any strict study procedures, which raise concerns about the project’s reproducibility. However, our goal was to create a system to improve the efficiency of our prescreening process and the developed sBPA system served that purpose. Additionally, there is a challenge of limited generalizability of findings from this quality-improvement project, as it applies to other settings. This method is specific to the Epic Reporting Workbench in an integrated delivery network of hospitals; therefore, the study staff would be required to use the Epic Reporting Workbench to test the sBPA’s success in reducing screening time and expanding recruitment. Moreover, information bias may be a problem, because the traditional screening method was performed by one research coordinator who then checked the in-basket messages to compare the patients identified from both methods. With adequate resources, these procedures would ideally be carried out by at least two different screening methods. Although the research team conceived the idea to setup the sBPA system, we depended on another team for actual development and associated timelines. There is also a cost associated with the development, to pay for the developers’ efforts. Thus, researchers would need to account for these costs in their study budget.

### Conclusions

Utilizing EHR for clinical research and automation of the recruitment workflow process has broad implications for accelerating innovation in health care. The sBPA notifications can help reduce the amount of time spent screening and increase the potential patient pool for study recruitment, resulting in increased cost-efficiency and accelerated study-completion timelines.

## Appendix

Multimedia Appendix 1 An example of a pop-up notification that could be used to inform clinical decision making. To clear this notification, an action is required from the provider.

Multimedia Appendix 2 All institutional review board–approved research staff receive a best practice alert, real-time notification in their Epic in-basket. This is easily accessed under the "BestPractice" tab in the lower left-hand corner of the Epic homepage.

Multimedia Appendix 3 The silent Best Practice Alert notifications detect an event of interest (in this case, hospital admissions), using criteria preselected by the Emerald-COPD research staff. COPD: chronic obstructive pulmonary disorder.

Multimedia Appendix 4 From in-basket view, the research staff can identify patient demographics and reasons for potential eligibility.

Multimedia Appendix 5 The study staff can directly access the “Encounter” tab for further information regarding patient eligibility.

## Abbreviations

BPA: Best Practice Alert
COPD: chronic obstructive pulmonary disease
EHR: electronic health record
EHR4CR: Electronic Health Records for Clinical Research
ICD-10: International Statistical Classification of Diseases and Related Health Problems 10th revision
PCOR: Patient-Centered Outcomes Research
PeRC: Partners eCare Research Core
REDCap: Research Electronic Data Capture
sBPA: silent Best Practice Alert
