# Supplementary material for: Use of Electronic Health Records to Develop and Implement a Silent Best Practice Alert Notification System for Patient Recruitment in Clinical Research: Quality Improvement Initiative
Source: JMIR Med Inform. 2019 Apr 26;7(2):e10020. doi: 10.2196/10020 (PMC6658304; doi:10.2196/10020)
Supplement: Multimedia Appendix 1 [file medinform_v7i2e10020_app1.pdf]

BestPractice Advisory - laqbpa,Opioid

Important (Advisory: 1)

Opioid medication management agreement not identified in eCare system. If the opioid you are prescribing is for chronic use, please complete and file an Opioid Agreement.

Opioid Medication Management Agreement (Blank Form)

Acknowledge Reason

Will complete & file agreement | Agreement previously completed/filed | Not indicated at this time

Accept & Stay | Accept | Dismiss
