# Supplementary material for: Use of Electronic Health Records to Develop and Implement a Silent Best Practice Alert Notification System for Patient Recruitment in Clinical Research: Quality Improvement Initiative
Source: JMIR Med Inform. 2019 Apr 26;7(2):e10020. doi: 10.2196/10020 (PMC6658304; doi:10.2196/10020)
Supplement: Multimedia Appendix 2 [file medinform_v7i2e10020_app2.pdf]

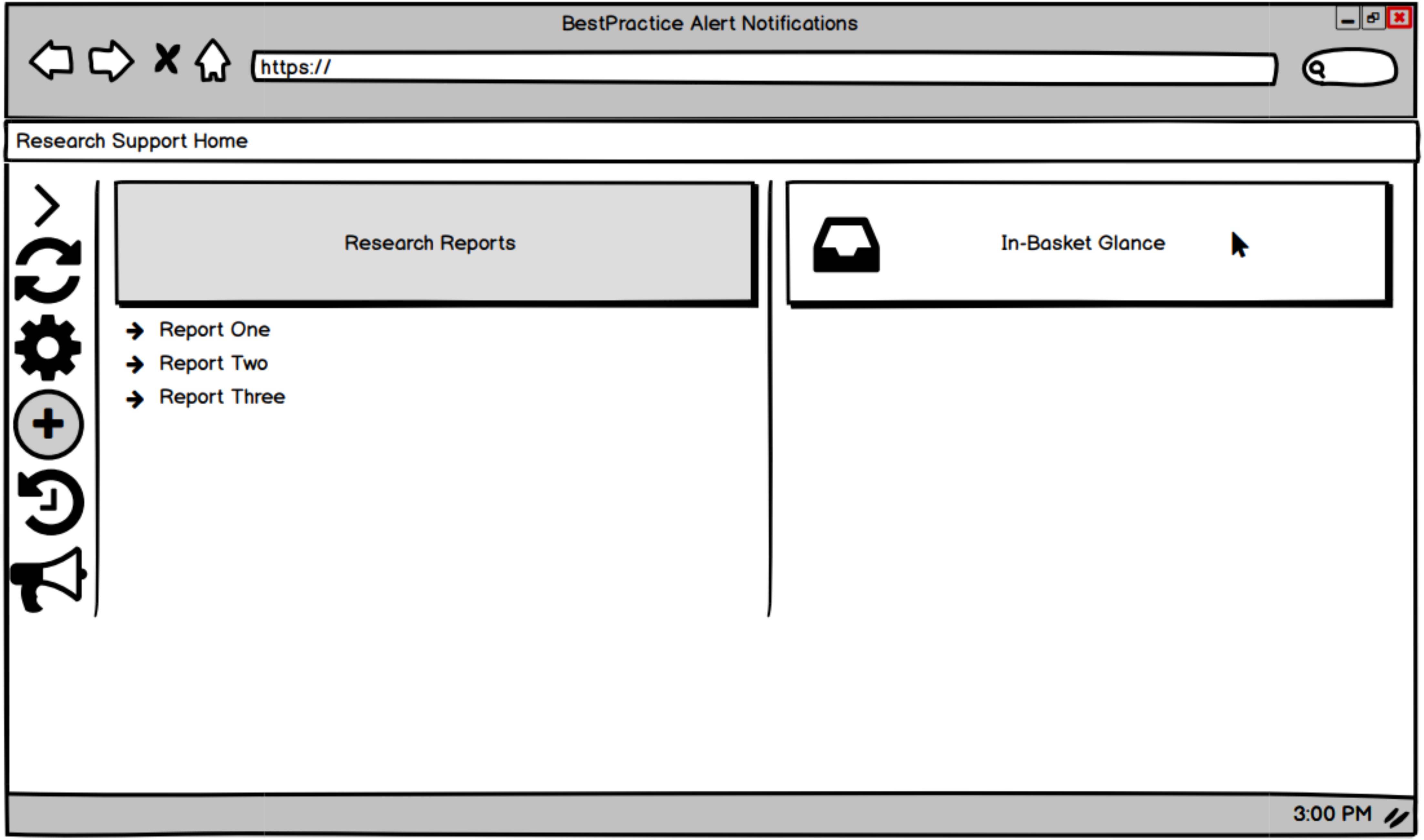
BestPractice Alert Notifications
https://
Research Support Home
Research Reports
Report One
Report Two
Report Three
In-Basket Glance
3:00 PM
