# Supplementary material for: Use of Electronic Health Records to Develop and Implement a Silent Best Practice Alert Notification System for Patient Recruitment in Clinical Research: Quality Improvement Initiative
Source: JMIR Med Inform. 2019 Apr 26;7(2):e10020. doi: 10.2196/10020 (PMC6658304; doi:10.2196/10020)
Supplement: Multimedia Appendix 3 [file medinform_v7i2e10020_app3.pdf]

BestPractice Alert Notifications

https://

Research Support Home

823

Messages

Research Notifications

Best Practice Alerts

Status	Msg Date	Msg Time	Subject	Patient	Age	DoB	Page
Unread	01/01/20	12:00PM	NEW A	Patient, Test	00	01/01/1950	
Unread	01/01/20	12:00PM	NEW A	Patient, Test	00	01/01/1950	
Unread	01/01/20	12:00PM	NEW A	Patient, Test	00	01/01/1950	
Unread	01/01/20	12:00PM	NEW A	Patient, Test	00	01/01/1950	
Unread	01/01/20	12:00PM	NEW A	Patient, Test	00	01/01/1950	
Unread	01/01/20	12:00PM	NEW A	Patient, Test	00	01/01/1950	
Unread	01/01/20	12:00PM	NEW A	Patient, Test	00	01/01/1950	
Unread	01/01/20	12:00PM	NEW A	Patient, Test	00	01/01/1950	
Unread	01/01/20	12:00PM	NEW A	Patient, Test	00	01/01/1950	
Unread	01/01/20	12:00PM	NEW A	Patient, Test	00	01/01/1950	
Unread	01/01/20	12:00PM	NEW A	Patient, Test	00	01/01/1950	
Unread	01/01/20	12:00PM	NEW A	Patient, Test	00	01/01/1950	
Unread	01/01/20	12:00PM	NEW A	Patient, Test	00	01/01/1950	

3:00 PM
