# Supplementary material for: Use of Electronic Health Records to Develop and Implement a Silent Best Practice Alert Notification System for Patient Recruitment in Clinical Research: Quality Improvement Initiative
Source: JMIR Med Inform. 2019 Apr 26;7(2):e10020. doi: 10.2196/10020 (PMC6658304; doi:10.2196/10020)
Supplement: Multimedia Appendix 4 [file medinform_v7i2e10020_app4.pdf]

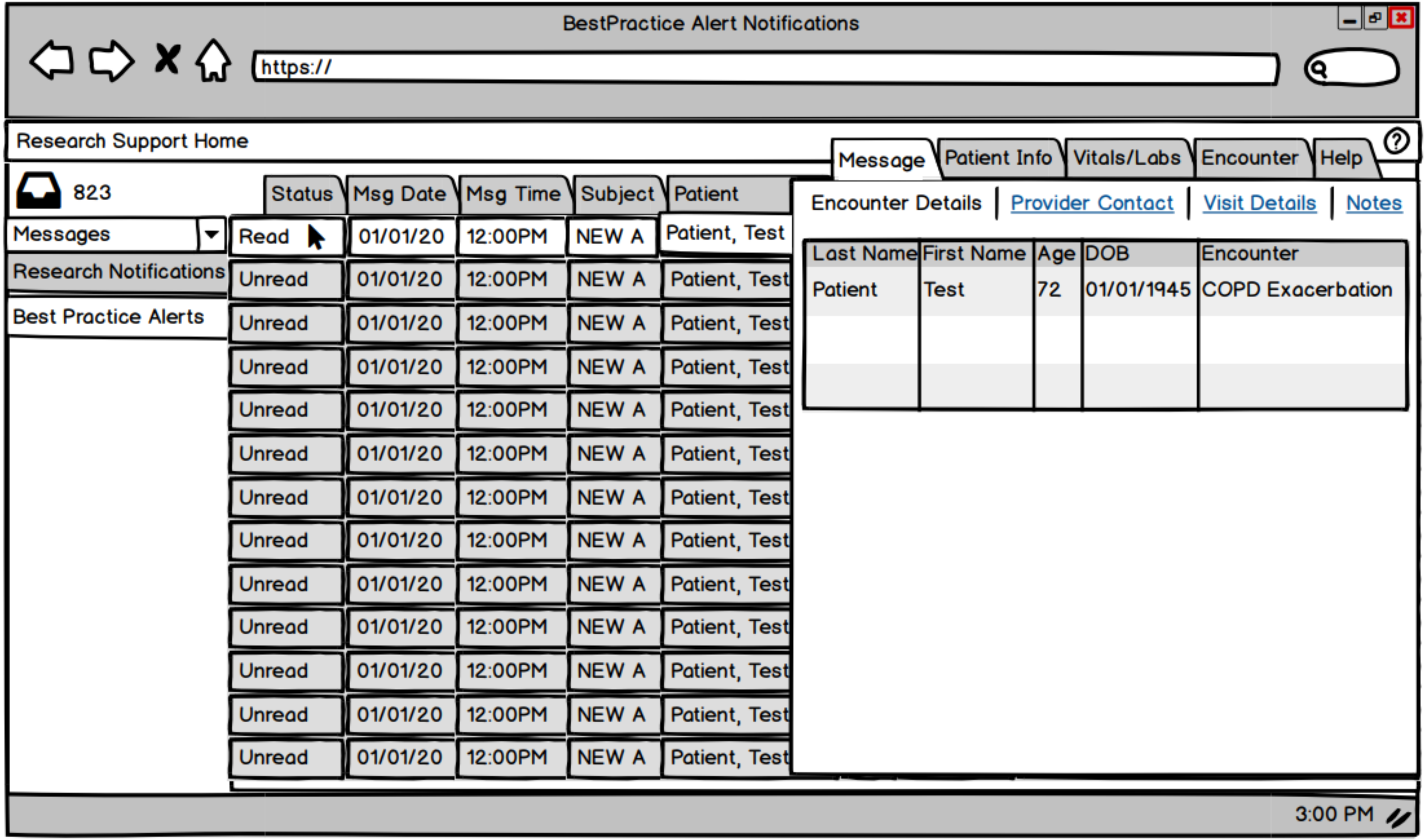
BestPractice Alert Notifications
https://
Research Support Home
Message
Patient Info
Vitals/Labs
Encounter
Help
823
Status
Msg Date
Msg Time
Subject
Patient
Messages
Research Notifications
Best Practice Alerts
Read 01/01/20 12:00PM NEW A Patient, Test
Unread 01/01/20 12:00PM NEW A Patient, Test
Unread 01/01/20 12:00PM NEW A Patient, Test
Unread 01/01/20 12:00PM NEW A Patient, Test
Unread 01/01/20 12:00PM NEW A Patient, Test
Unread 01/01/20 12:00PM NEW A Patient, Test
Unread 01/01/20 12:00PM NEW A Patient, Test
Unread 01/01/20 12:00PM NEW A Patient, Test
Unread 01/01/20 12:00PM NEW A Patient, Test
Unread 01/01/20 12:00PM NEW A Patient, Test
Unread 01/01/20 12:00PM NEW A Patient, Test
Unread 01/01/20 12:00PM NEW A Patient, Test
Unread 01/01/20 12:00PM NEW A Patient, Test
Encounter Details
Provider Contact
Visit Details
Notes
Last Name First Name Age DOB Encounter
Patient Test 72 01/01/1945 COPD Exacerbation
3:00 PM
