# Supplementary material for: Use of Electronic Health Records to Develop and Implement a Silent Best Practice Alert Notification System for Patient Recruitment in Clinical Research: Quality Improvement Initiative
Source: JMIR Med Inform. 2019 Apr 26;7(2):e10020. doi: 10.2196/10020 (PMC6658304; doi:10.2196/10020)
Supplement: Multimedia Appendix 5 [file medinform_v7i2e10020_app5.pdf]

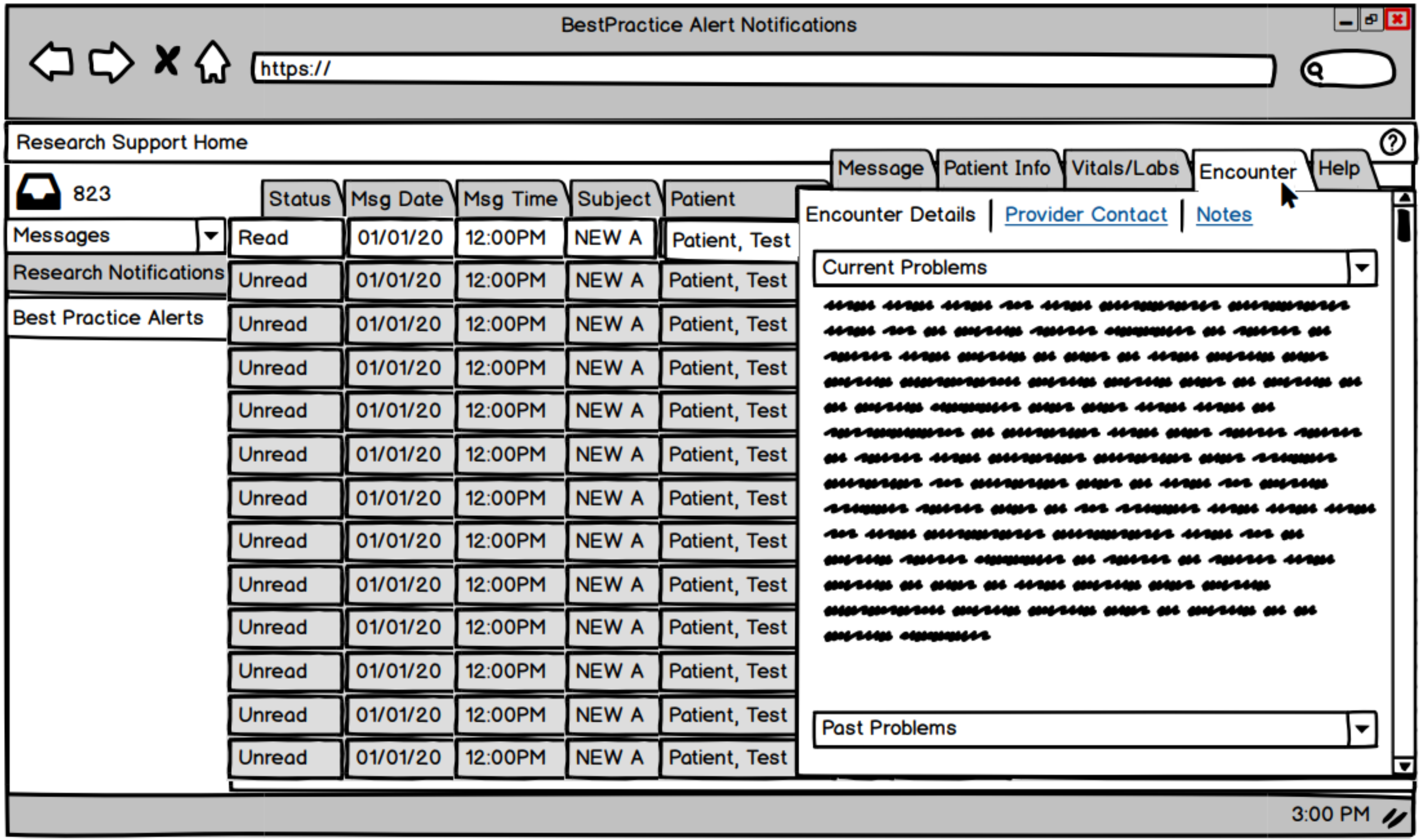

BestPractice Alert Notifications
https://
Research Support Home
823
Messages
Research Notifications
Best Practice Alerts
Status
Msg Date
Msg Time
Subject
Patient
Read
01/01/20
12:00PM
NEW A
Patient, Test
Unread
01/01/20
12:00PM
NEW A
Patient, Test
Message
Patient Info
Vitals/Labs
Encounter
Help
Encounter Details
Provider Contact
Notes
Current Problems
Past Problems
3:00 PM
